# When high work engagement is negative for family tasks: mechanisms and boundary conditions

**DOI:** 10.3389/fpsyg.2024.1403701

**Published:** 2024-06-27

**Authors:** Ike E. Onyishi, Christoph Nohe, Fabian O. Ugwu, Lawrence O. Amazue, Guido Hertel

**Affiliations:** ^1^Department of Psychology, University of Nigeria, Nsukka, Nigeria; ^2^Chair of Organizational & Business Psychology at the University of Münster, Münster, Germany; ^3^Department of Psychology, Alex Ekwueme Federal University, Ndufu-Alike, Nigeria

**Keywords:** work engagement, organizational citizenship behavior, rumination, work-family conflict, Nigeria

## Abstract

**Background:**

Typically, work engagement is positively related to beneficial job outcomes. Earlier studies, however, revealed a “dark side” of work engagement showing negative effects such as more work-family conflict. Using a resource perspective, our study seeks to better understand *why* and *when* these negative effects of work engagement occur. Specifically, we test a new model in which the relationship of work engagement with work-family conflict is mediated by organizational citizenship behavior (OCB) and work rumination. Moreover, we argue that employees’ resource-building strategies (i.e., job crafting) and resource levels (i.e., psychological capital) buffer resource depletion due to high work engagement.

**Methods:**

We tested our assumptions in a field study that involved data collected on three measurement points with 523 employees from Nigeria. The measures consist of Utrecht Work Engagement Scale, Organizational Citizenship Behavior Scale, Work Rumination Scale, Psychological Capital Scale, Job Crafting Measure, Work-family Conflict Scale, and demographic variables. Structural Equation Modeling (SEM) was used to test the hypotheses.

**Results and discussion:**

Results from latent structure equation modelling confirm that work rumination mediates the positive relationship between work engagement and work-family conflict. Additionally, our findings suggest that behavioral engagement (i.e.,OCB) and work rumination mediate the relationship between work engagement and work-family conflict. Moreover, psychological capital mitigated the relationships of work engagement with work rumination, but not job crafting. Our study helps to better understand the “dark side” of work engagement and offers implications on how to mitigate its detrimental relationship with work-family conflict.

## Introduction

Usually, work engagement – a positive-motivational state characterized by vigor, dedication, and absorption (Schaufeli et al., 2002; Saks and Gruman, 2014) – is beneficial to employees and employers (e.g., van Zyl et al., 2021; Hsieh and Kao, 2022). Indeed, meta-analyses have contributed to this consensus, demonstrating that work engagement is related to positive individual and organizational outcomes like high performance rating, job satisfaction, organizational citizenship behavior (OCB), and work commitment (e.g., Christian et al., 2011; Borst et al., 2020; Sari et al., 2020; Neuber et al., 2022; Yildiz and Yildiz, 2022).

However, studies revealed negative effects and found that work engagement is associated with difficulties combining work and family roles (Halbesleben et al., 2009), a phenomenon typically called work–family conflict (Carlson et al., 2000). Researchers (e.g., Bakker et al., 2011) who call for an investigation on the reverse or “bad” side of engagement reasoned that there could be a limit to engagement because of limited resources available to an individual. Recent surprising evidence shows that work engagement is also related to several negative consequences that tend to threaten the organization and its members including higher sympathetic activation, exhaustion, and high work–family conflict (e.g., Halbesleben et al., 2009; Baethge et al., 2021; Junker et al., 2021). Work engagement is equally found to have a U-shaped, or curvilinear relationship with psychological distress (Shimazu et al., 2018) and job performance (Bouckenooghe et al., 2022) demonstrating that high level of engagement can be detrimental to both the individual and the organization. In addition, several studies (e.g., Parkes and Langford, 2008; Shankar and Bhatnagar, 2010) support the link between work engagement and work–life/family constructs. If employees experience a lack of resources (i.e., depletion of role resources; Rothbard, 2001), they will find it difficult to fulfill their family responsibilities, thus creating work–family conflict (Chernyak-Hai and Tziner, 2016).

As George (2011) argues, employees who are highly engaged are likely to have diminished time and energy resources to deal with roles outside of work and may also sacrifice other aspects of their lives to sustain their high engagement at work. Although earlier studies addressed the association of work engagement with work–family conflict (Halbesleben et al., 2009), *why* and *when* this detrimental relationship occurs is not well understood. Examining the mechanisms of the “dark side” holds the potential for an improved psychological and behavioral understanding of work engagement. Additionally, a better understanding of when negative effects occur can help to mitigate the detrimental relationship of work engagement with work–family conflict.

**Figure 1:**
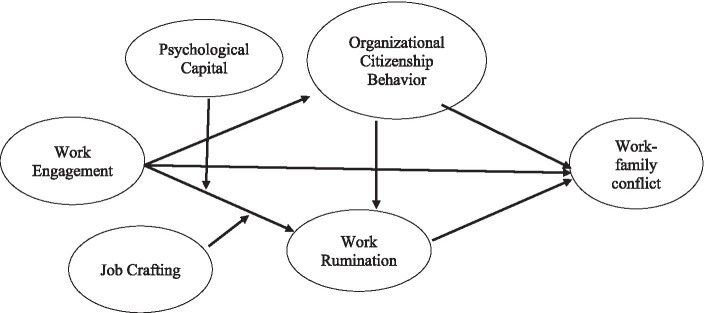
Study model.

Building on the Conservation of Resources (COR: Hobfoll, 2011) and Work-Home Resources (W-HR: ten Brummelhuis and Bakker, 2012) perspectives and prior work on work engagement (Shimazu et al., 2018), we argue that there are two possible pathways through which engagement may have negative effects on work–family conflict. The first is a behavioral pathway whereby high work engagement leads to excessive amounts of time and effort at work (Beckers et al., 2004; Halbesleben et al., 2009), and the second is a cognitive pathway whereby high work engagement leads to a continuously high level of arousal and activation (Shimazu et al., 2018; Baethge et al., 2021) that inhibit recovery processes. Based on the primacy of loss and resource investment principles of the COR model (Hobfoll et al., 2018), we argue that high work engagement may lead to employees’ investment of resources such as cognitive, emotional, and physical energy (Rich et al., 2010) in extra-role activities such as OCB (Christian et al., 2011; Matta et al., 2015; Smith et al., 2020). More specifically, we assume that OCB and rumination about work are manifestations of high levels of concentration and should mediate the association of work engagement with work–family conflict. A previous study has already addressed the mediating role of OCB (Halbesleben et al., 2009), however, work rumination has not been tested in prior research. We also reason that engagement in OCB during work hours may also lead to rumination after work because proactive work behavior such as OCB is a resource-intensive activity which causes irritability and may result in work-related rumination (Pingel et al., 2019). For instance, engaging in OCB involves additional effort or sacrifice beyond an employee’s job requirements and may include working longer periods (van Zyl et al., 2021; Chiaburu et al., 2022; Ma et al., 2022) and difficulties in completing job tasks (e.g., Bolino and Turnley, 2005). This situation may lead to mental preoccupation with job-related issues after work (Watkins, 2008). The inability to detach from work can increase the likelihood of rumination as individuals struggle to switch off their work-related thoughts and worries (Sonnentag and Niessen, 2020; Tuerktorun et al., 2020; Weigelt et al., 2023). Investing emotional and cognitive resources in ruminating about work may further deplete the resources required to function at home. As resources available to individuals are finite, investment of cognitive and emotional resources in ruminating about work after work period can result in further resource loss, leading to work–family conflict. Additionally, we argue that employees’ resource-building strategies (i.e., job crafting) and resource levels (i.e., psychological capital) buffer the detrimental relationship between work engagement and work–family conflict. To the best of our knowledge, prior studies did not examine mitigating factors in work engagement–work–family conflict relationships. Our study model is presented in Figure 1.

We make important contributions to the literature. The present study adds to the small but growing literature that focuses on the negative effects or “the dark side” of work engagement, thereby responding to the consistent calls for more research in the area (Bakker et al., 2011). Specifically, by examining work rumination as a potential mediator, we examine a cognitive resource perspective that explains how work engagement might lead to work–family conflict. Whereas prior research has concentrated on a behavioral resource investment mechanism (Halbesleben et al., 2009; Smith et al., 2020), our approach provides a broader perspective integrating both behavioral and cognitive resource views. By testing resources-related moderators in our study, we advance the understanding of how personal resources (psychological capital) as well as resource creation and conservation strategies (job crafting) might serve as boundary conditions for the detrimental effect of work engagement. In addition, the study involved full-time workers selected from a variety of organizations in Southeast Nigeria. The study therefore is one of the few attempts at understanding work engagement and work–family conflict in a cultural environment (Nigerian work environment) different from North America, Europe, and Asia.

### Hypotheses development

#### Work engagement and work–family conflict: mediating role of OCB

It is established that individuals’ involvement in work roles can interfere with the fulfillment of family roles, which is typically called work–family conflict (Frone et al., 1992). We argue that work–family conflict can occur, at least in part, because of high levels of employee work engagement. Work engagement is defined as “a positive, fulfilling, affective-motivational state of work-related well-being” that comprises vigor, dedication, and absorption (Leiter and Bakker, 2010, p. 1).

The COR theory (Hobfoll, 2011) and the W-HR model (ten Brummelhuis and Bakker, 2012) provide a lens to better understand the relationship between work engagement and work–family conflict. Specifically, work engagement entails excessive involvement in job roles that consumes personal resources, such as time and physical or mental energy (Bolino et al., 2015). The WH-R theory suggests that volatile resources such as time and energy are fleeting, and as such if they are used cannot be available for other purposes (ten Brummelhuis and Bakker, 2012).

Specifically, excessive involvement in one’s role consumes personal resources, leaving insufficient resources to allocate to other roles. Thus, engaged employees during work periods devote a lot of resources (time, energy) to their jobs and in so doing deplete the resources they require to cope with family roles and may experience work–family conflict. Previous studies have shown that individuals who experience resource depletion due to engagement in work activities experience work–family conflict. For instance, Oren and Levin (2017) found that individuals who reported a higher threat of loss of resources reported experiencing higher work–family conflict than those who reported resource enrichment. Moreover, reports of previous studies (e.g., Pak et al., 2022; Brandão and Matias, 2024; Dishon-Berkovits et al., 2024; Li et al., 2024) have consistently shown that personal and job resources are important in reducing work–family conflict and depletion of such resources lead to the experience of work–family conflict. In sum, the COR and W-HR views suggest that work engagement is positively related to work–family conflict because work engagement involves resource investment during work (e.g., time, energy) and the individual is left with depleted resources and unable to effectively perform family roles after work.

It has been observed that engaged employees are enthusiastic about their work, identify highly with their organization, and are immersed in their work activities (Schaufeli et al., 2002). Work engagement also entails being highly absorbed in work or dedicated to one’s work. This suggests that time resources are being used in the work domain with potentially less for other domains. Bakker et al. (2011) had earlier hypothesized that the absorption component of work engagement has the potential to produce unhealthy behavior in life domains outside of work because individuals who are highly absorbed or immersed in their work may tend to neglect other non-work responsibilities. As absorption is viewed as both a component of workaholism and work engagement, its effects on resource depletion appear obvious. Absorption in work refers to “being fully concentrated and deeply engrossed in one’s work, whereby time passes quickly, and one has difficulties with detaching oneself from work” (Schaufeli et al., 2002, p. 75). Because of their high levels of absorption and dedication at work, engaged employees have also been reported to be involved in extra-role behaviors (Halbesleben et al., 2009; Smith et al., 2020) more than less engaged colleagues.

Employees’ work engagement fuels behavioral manifestations in terms of engaging in extra-role behavior such as OCB (Halbesleben et al., 2009). OCB can be considered a behavioral manifestation of work engagement because those behaviors are discretionary but effective in the functioning of organizations (Bolino et al., 2015). Indeed, prior studies continuously report positive relationships between work engagement and OCB (e.g., Halbesleben et al., 2009; Babcock-Roberson and Strickland, 2010; Alfes et al., 2013; Demerouti et al., 2015; Matta et al., 2015; Gupta et al., 2017; Smith et al., 2020). Engaging in OCB, like task performance, requires high employee concentration and investment of resources in performing extra job responsibilities (Wu et al., 2023). OCB can be conceptualized as one of the job performance dimensions (e.g., Ng and Feldman, 2008). Specifically, behaviors such as helping a coworker or going beyond minimum standards are likely to require employees’ resources such as time and energy.

Additionally, OCB entails that employees take a larger workload and therefore represent resources-intensive contributions to influence job outcomes (Bolino et al., 2015; Trougakos et al., 2015; Breevaart et al., 2020). Individuals who engage in OCB may therefore find it more challenging to cope with non-job activities after work resulting in work–family conflict (ten Brummelhuis and Bakker, 2012; Deery et al., 2017; Germeys et al., 2019; Bolino et al., 2023; Chaudhary et al., 2023). To the best of our knowledge, only two studies (Halbesleben et al., 2009; Smith et al., 2020) have examined OCB as a mediator in the relationship between work engagement and work interference with family, suggesting that there is a need to constructively replicate the behavioral investment perspective with different samples and in other contexts (Hüffmeier et al., 2016). We replicate these earlier studies based on our reasoning that behavioral engagement tasks individual resources. We state:

*Hypothesis 1*: Work engagement positively relates to work–family conflict.

*Hypothesis 2*: OCB mediates the positive relationship between work engagement and work–family conflict, such that highly engaged workers report increased work–family conflict due to their high OCB.

#### Mediating role of work rumination

In addition to the mediating role of OCB, the study examines the mediating role of work rumination on the relationship between work engagement and work–family conflict. Rumination about work comprises perseverative thinking about work-related issues and events (Querstret and Cropley, 2012). This implies that employees are unable to switch off from work-related thoughts after work periods (Cropley et al., 2006). For example, employees may ruminate about uncompleted tasks, unresolved problems, or about upcoming work events (Querstret and Cropley, 2012). We propose that work rumination will mediate the relationship between work engagement and work–family conflict. Work engagement is a state whereby an employee has resources (e.g., energy) that exceed their job demands and this helps the individual to perform positive job behaviors (Simbula and Guglielmi, 2013). However, we argue that work engagement is also likely to increase work rumination. Specifically, employees high in work engagement are highly absorbed in their work (Schaufeli et al., 2002) and engage in many activities at work (Halbesleben et al., 2009) and might therefore continue to think about work even after leaving the workplace.

It is also likely that employees who exhibit high OCB will ruminate more about work after leaving the workplace because of the high level of activation occasioned by high involvement or engagement at work. Going the extra mile in performing job roles does not necessarily mean that one will accomplish all job tasks. Engaging in extra roles may expand an individual’s perception of work scope and lead to the inability to finish all work tasks before leaving the workplace (Bolino and Turnley, 2005). Engagement in OCB involves taking on additional work activities including performing work roles of other organizational members and may involve working longer periods (van Zyl et al., 2021; Chiaburu et al., 2022; Ma et al., 2022) and experience of difficulties in completing job tasks (e.g., Bolino and Turnley, 2005). Unaccomplished or unresolved goals increase the accessibility of goal-relevant information (Martin and Tesser, 1989; Brunstein and Gollwitzer, 1996; Querstret and Cropley, 2012) and trigger a mental preoccupation with the unresolved issues at hand (Watkins, 2008). These negative outcomes imply that OCB may be a behavioral manifestation of work engagement. Thus, instead of engaging in activities that help in the recovery of lost resources, engaged employees who have devoted their time performing OCB tend to get preoccupied with work-related issues at home and continue to ruminate about work after work periods. As rumination has been shown to prolong work-related activation and the resource-depleting effect of work stressors after work periods (Baethge et al., 2021), it leads to further depletion of available resources required to function effectively at home. According to the COR theory (Hobfoll, 2011; Hobfoll et al., 2018) and W-HR (ten Brummelhuis and Bakker, 2012), individuals strive to maintain resource levels and avoid resource loss and resources gained in one domain (work or home) can positively or negatively influence experiences and performance in the other domain. The interplay among work engagement, OCB, rumination, and work–family conflict can create negative effect in the long run. For instance, work engagement may initially lead to increased resource accumulation in the work domain, and the accumulated resources can be reinvested in performing extra role behavior such as OCB but if this is accompanied by rumination after work, the individual may have fewer resources available to fulfill family responsibilities. Integrating behavioral and cognitive resource perspectives into our model, we expect that work engagement will be positively related to behavioral engagement (indicated by OCB), which, in turn, will further task individual cognitive resources (indicated by work rumination), which will be related to work–family conflict. We state:

*Hypothesis 3*: Work rumination mediates the positive relationship between work engagement and work–family conflict.

*Hypothesis 4*: OCB and work rumination sequentially mediate the positive relationship between work engagement and work–family conflict, such that both increased OCB and elevated levels of work rumination are pathways through which high work engagement is associated with increased work–family conflict (i.e., work engagement → OCB → work rumination → work–family conflict).

#### Moderating effects of job crafting

While work engagement is likely to deplete employees’ resources, we argue that employees’ resource-building strategies should protect employees against resource depletion through work engagement. Our expectation is grounded in COR’s proposition that individuals must invest resources to protect them against resource loss and gain new resources (Hobfoll, 2011).

A strategy for building job resources is job crafting which refers to self-initiated changes that employees adopt in improving their personal or work goals (Wrzesniewski and Dutton, 2001; Tims et al., 2012). Job crafting is captured from four independent dimensions which focuses on increasing structural job resources, increasing social job resources, increasing challenging job demands, and decreasing hindering job demands (Tims et al., 2012). Structural job resources refer to issues such as resource variety, opportunity for development, and autonomy, while social job resources deal with social support, supervisory coaching, and feedback that enable the employee to perform his or her job more effectively (Tims et al., 2012). Thus, job crafting builds and conserves resources by seeking resources, seeking challenges, and reducing demands (Petrou et al., 2012).

Job crafting has been reported to be important in helping employees find a balance between job demands and resources to enhance person-job fit (Tims et al., 2012), and may serve as a moderator in this relationship (Zhang and Parker, 2019; Du Toit et al., 2022). Job crafting has been tested as a moderator in the relationships between perceived overqualification and job boredom (Sánchez-Cardona et al., 2020), job demands and burnout (Hakanen et al., 2017), job demands and work engagement (Hakanen et al., 2017), work engagement and team performance (Mäkikangas et al., 2016), and overqualification and turnover intention (Debus et al., 2020). The moderating effect of job crafting on the relationship between work engagement and job performance has been demonstrated in a meta-analysis (Oprea et al., 2019). Despite these attempts to justify the moderating capability of job crafting behavior, none examined its effect in the relationship between work engagement and work rumination.

We argue that the resource conservation and acquisition through job crafting should buffer resource depletion through work engagement. Conservation of resources occurs through job crafting because less time, physical, and cognitive resources are spent in performing work roles. Acquisition of additional resources occurs, for example, through actively changing working conditions. For instance, actively seeking social support from coworkers or supervisors can help employees build job resources through acquisition of new skills and/or can lead to conservation of resources by sharing of job responsibilities. Through successful job crafting employees are likely going to leave the work environment with enough resources to deal with other life demands and may ruminate less about work during non-work periods. Empirical studies (e.g., Tims et al., 2013) have supported the resource accumulation and conservation mechanisms of job crafting suggesting that individuals who craft their jobs may be protected from adverse resource loss during work periods. Thus, when job crafting counteracts resource loss due to work engagement, employees may end up with more resources indicated by less work rumination. We state:

*Hypothesis 5*: Job crafting moderates the positive relationship between work engagement and work rumination in such a way that this relationship is weaker when job crafting is high than when it is low.

#### Moderating effects of psychological capital

In addition to resource building strategies, dispositional differences in personal resources are likely to influence the relationship between work engagement and work rumination. Our expectation follows from COR’s proposition that individuals with greater resources are less vulnerable to resource loss (Hobfoll, 2011). Core aspects of personal resources are covered by the concept of psychological capital, comprising self-efficacy, optimism, hope, and resilience (Luthans et al., 2007). Based on the COR theory (Hobfoll, 2011) we reason that psychological capital can serve as a resource and reservoir from which employees can draw in times of need. As such, individuals with high psychological capital positively appraise work situations, actively cope with work demands, and generally have improved psychological well-being and adjustment both at the short and long term (Avey et al., 2010).

As earlier noted, individuals who are highly engaged are more likely to go the extra mile in performing work roles (Halbesleben et al., 2009; Alfes et al., 2013; Smith et al., 2020) and may continue to ruminate about work after work periods due to high levels of activation and pre-occupation with work-related thoughts (Baethge et al., 2021). This may predispose them to depletion of energy resources required to perform home-related activities. However, individuals who have high psychological capital have confidence in their ability to perform different tasks, believe that they can achieve set goals through different means, perceive life events more positively, and have the capacity to recover quickly from resource loss. This means that individuals with high psychological capital will have more adequate resources to face other life challenges even after devoting enormous cognitive, time and energy resources at work and after work (Luthans et al., 2010). We argue that psychological capital can serve as a personal resource that curbs potential negative effects of work engagement on rumination.

*Hypothesis 6*: Psychological capital moderates the relationship between work engagement and work rumination such that highly engaged workers are less likely to ruminate at work when their psychological capital is high compared to those with low psychological capital.

## Methods

### The Nigerian context

Nigeria presents an interesting setting to study the relationship between work behavior and family life. Earlier studies (House et al., 2004) classified Nigeria to be high in collectivism and power distance and low in performance orientation when compared with most other countries in North America and Europe. These differences may have implications for work behaviors and family life. For example, scholars (e.g., Onyishi and Ogbodo, 2012; Onyishi et al., 2020) observed that employees in Nigeria appear not to engage in proactive work behaviors and have poor attitudes to work (Onyishi et al., 2022). Again, as a highly collectivists culture, family life is highly valued in Nigeria (Ugwu et al., 2019) suggesting that there may be high tendencies for workers to experience work–family conflict. Yet, there are paucity of data on work and family interface in Nigeria in comparison to North America and Europe where most of the studies on work-family relations were conducted (Amazue and Onyishi, 2016).

The economic and business globalization has also made work-family issues increasingly important in both developed and developing countries (Reddy et al., 2010; Flood and Genadek, 2016). Extending research that is nearly exclusively based on American, Asian, and European samples (e.g., Gallie and Russell, 2009; Shih et al., 2010; Wang et al., 2010; Yalabik et al., 2015), to other contexts is necessary.

### Participants and procedure

The participants in the study were 523 full-time working adults in a variety of organizations including universities, banks, and hospitals in Southeast region of Nigeria. The inclusion criteria for participation were that the participant had worked for a minimum of 1 year, above 18 years, live with at least one family member at the time of the study, and volunteered to participate in the study. Among the participants 50.86% were men. The mean age was 37.08 (*SD* = 8.08) years with 67.37% of them being married. On average, participants reported having 2.10 children (*SD* = 2.16). They have been employed in their current job for an average of 7.86 years (*SD* = 6.40). Ten graduate students of psychology collected the data as part of their research experience assignment. The graduate students who served as research assistants recruited participants from work organizations operating within south-east region of Nigeria. The research assistants approached the participants with an introduction letter that included information on the study seeking participants’ consent to take part in the study. A total of 587 participants who agreed to participate in the study were first handed over the study booklet for Time 1 survey by the research assistants and they followed up on the same set of participants that they recruited at Time 1 for the Time 2 and Time 3 surveys to collect the completed questionnaire directly from them. The participants completed the English version of the survey. At Time 1, we collected data on demographic variables, work engagement, and moderators (job crafting, psychological capital). At approximately one-month intervals, we collected data on OCB and work rumination, for the Time 2 surveys. At Time 3 (approximately 3 months after Time 2 data collection) we collected data on work–family conflict.

We assume that our time intervals of 1 month between T1 and T2 and 3 months after Time 2 data collection are reasonable because smaller measurement intervals or inclusion of multiple waves have greater power to detect an effect and lead to more accurate estimates of population parameters (Taris and Kompier, 2014). Moreover, several meta-analytic studies (e.g., Riketta, 2008) indicated that effects in panel studies wear away as the time lag between two measurements increases.

The research assistants at the various collection times were responsible for handing over of the study booklets to the participants and the collection of completed surveys (in a sealed envelope) from the participants they recruited. Participants did not receive any incentive for taking part in the study. The study was approved by the Research Ethics Committee of the Department of Psychology, University of Nigeria, Nsukka.

At the end of Time 1, we had 575 usable data. At T2, 42 people dropped out. These dropouts were older [*M* = 40.67, *SD* = 8.29 vs. *M* = 37.08, *SD* = 8.08; *t*(562) = 2.76, *p* = 0.006], had a longer job tenure [*M* = 13.79, *SD* = 8.51 vs. *M* = 7.86, *SD* = 6.40; *t*(45) = 4.41, *p* < 0.001], and reported higher levels of work engagement [*M* = 4.52, *SD* = 0.81 vs. *M* = 4.18, *SD* = 1.07; *t*(53) = 2.57, *p* = 0.013] than non-dropouts. At T3, 10 people dropped out. Comparisons revealed no differences between dropouts’ and non-dropouts’ work engagement, OCB, gender, age, and job tenure, except that dropouts’ indicated to ruminate less [*M* = 3.83, *SD* = 1.61 vs. *M* = 4.80, *SD* = 1.44; *t*(519) = −2.00, *p* = 0.046]. Our final sample consisted of 523 participants (overall response rate of 89.10%).

### Measures

#### Work engagement

Work engagement was assessed with the short version of the Utrecht Work Engagement Scale (UWES-9; Schaufeli and Bakker, 2003). The items capture three dimensions of work engagement: vigor (e.g., At my job, I feel strong and vigorous.), dedication (e.g., My job inspires me), and absorption (e.g., I feel happy when I am working intensely). Items were scored on a 7-point rating scale ranging from 0 (never) to 6 (always). The reliability and validity of the UWES-9 has been established in previous research on Nigerian samples (Ugwu and Onyishi, 2020; Ugwu et al., 2023). Cronbach’s alpha of 0.87 was obtained for the present study.

#### Job crafting

Job crafting was assessed with the 21-item four-dimensional scale developed by Tims et al. (2012). The four dimensions were increasing structural job resources (e.g., ‘I try to develop my capabilities’), increasing social job resources (e.g., ‘I ask others for feedback on my job performance’), increasing challenging job demands (e.g., ‘I try to make my work more challenging by examining the underlying relationships between aspects of my job’), and decreasing hindering job demands (e.g., ‘I manage my work so that I try to minimize contact with people whose problems affect me emotionally’). Items were rated on a 5-point scale, ranging from 1 (‘never’) to 5 (‘very often’). The scale was reported to be reliable and valid in Nigerian samples (Arinze et al., 2022; Ujoatuonu et al., 2023). Cronbach’s alpha of 0.87 was obtained.

#### Psychological capital

Psychological capital was examined with the 24-item scale developed by Luthans et al. (2007) which captures the four components of psychological capital (efficacy, hope, resilience, and optimism). Sample items include: ‘I feel confident analyzing a long-term problem to find a solution,’ ‘If I should find myself in a jam at work, I could think of many ways to get out of it,’ ‘I can get through difficult times at work because I’ve experienced difficulty before,’ ‘I approach this job as if “every cloud has a silver lining.’ Items were rated on a 6-point scale, ranging from 1 (strongly disagree) to 6 (strongly agree). Luthans et al. (2007) reported Cronbach’s alpha of 0.84 for all items (*α* = 0.77 for efficacy, *α* = 0.73 for hope, *α* = 0.72 for resilience, and *α* = 0.66 for optimism). Previous research in Nigeria has also obtained acceptable reliability and validity for the scale (Ugwu et al., 2018; George et al., 2023). The Cronbach’s alpha was 0.84 for the current study.

#### Organizational citizenship behavior

Organizational citizenship behavior was measured with Williams and Anderson’s (1991) 14-item scale. The scale included two dimensions of OCB – OCB directed to specific individuals in the organization (OCBI), and OCB directed to the organization (OCBO). Sample items include: ‘I help others who have heavy workloads,’ ‘I assist supervisor with his/her work (when not asked)’. Items were rated on a 5-point scale, ranging from 1 (strongly disagree) to 5 (strongly agree). We excluded three items because they showed low standardized factor loadings (i.e., <0.25). Previous studies in Nigeria has reported good reliability and validity for the OCB sale (Ladebo, 2005; Onyishi, 2006). Overall, the scale had a Cronbach’s alpha of 0.84.

#### Work rumination

Work rumination was assessed with a 4-item measure earlier used by Cropley et al. (2006). Participants were asked to rate possible ways they may feel about their present job/work after they have ended the day’s work. The items are: (1) ‘Did you think about work?’ (2) ‘Did you think about future work?’ (3) ‘Did you think about things that had happened at work?’ (4) ‘Would you describe your work-related thoughts as repetitive/recurring?’ Participants were asked to rate their level of work rumination on a 7-point scale ranging from 1 (not at all) to 7 (all the time). This measure has adequate reliability and validity in Nigeria (Ugwu et al., 2014). Cronbach’s alpha was 0.87 was obtained for the current study.

#### Work–family conflict

Work–Family Conflict was assessed with 9 items from Carlson et al. (2000) work-family scale. Sample items are: ‘My work keeps me from my family activities more than I would like,’ ‘When I get home from work I am often too frazzled to participate in family activities/responsibilities,’ ‘The problem-solving behaviors I use in my job are not effective in resolving problems at home.’ Items were rated on a 5-point scale, ranging from 1 (strongly disagree) to 5 (strongly agree). This measure has adequate reliability and validity in Nigeria (Ugwu et al., 2014). For the present study, a Cronbach’s alpha of 0.83 was obtained.

### Analysis

We tested all hypotheses using Structural Equation Models (SEM) with latent variables. For work engagement, work–family conflict, OCB, job crafting, and psychological capital, we modeled second-order factors (i.e., items loaded on their respective facet and the facets loaded on their respective second-order factor). For work rumination, we modeled a first-order factor because work rumination does not have multiple facets (i.e., items loaded on a single-order factor). For our mediation Hypotheses 2, 3, and 4, we used the product-of-coefficients method to calculate the indirect effects (MacKinnon et al., 2002). For our moderation Hypotheses 5 and 6, we used latent moderated structural equation modeling (LMS; Klein and Moosbrugger, 2000). We conducted the analyses with Mplus 7.31 (Muthén and Muthén, 1998–2012) and full information maximum likelihood estimation.

## Results

We conducted a series of confirmatory factor analyses to examine the distinctiveness of the six constructs (i.e., work engagement, OCB, rumination, work–family conflict, psychological capital, and job crafting). Results revealed that the hypothesized six-factor model fitted the data satisfactorily [*χ*^2^ (2048) = 3,290.49, CFI = 0.90, TLI = 0.89, RMSEA = 0.03, SRMR = 0.05] and better as compared to five-factor models that combined work engagement and work–family conflict [*χ*^2^ (2053) = 3,554.64, CFI = 0.88, TLI = 0.87, RMSEA = 0.04, SRMR = 0.06; Δ*χ*^2^ (5) = 264.15, *p* < 0.01] or work engagement and OCB as a common factor [*χ*^2^ (2053) = 3,468.39, CFI = 0.88, TLI = 0.88, RMSEA = 0.04, SRMR = 0.06; Δ*χ*^2^ (5) = 177.90, *p* < 0.01]. RMSEA and SRMR exceeded the commonly used cutoff values of 0.06 and 0.08 (Hu and Bentler, 1999), whereas CFI and TLI were only close to 0.95. A possible reason is that Hu and Bentler (1999) used a model in their simulation study that differed from our model in important characteristics that influence cutoff values, such as number of items and factors, model type, magnitude of the standardizes loadings and factor reliability. Indeed, methodologists have repeatedly cautioned against the over generalizability of fixed cutoffs derived from a single simulation whose conditions only represent a disparate model subspace (McNeish and Wolf, 2023). Therefore, we proceeded with our hypothesized six-factor model and concluded that our measures captured distinct constructs.

Table 1 shows means, standard deviations, and correlations among the study variables. Notably, work engagement and work–family conflict are positively correlated (*r* = 0.49) which preliminarily supports Hypothesis 1.

**Table 1:** Means, standard deviations, and correlations among the study variables.

	Mean	*s.d.*	1	2	3	4	5	6	7	8	9	10
1. Work engagement	4.18	1.07	–									
2. WFC	3.20	0.68	0.49***	–								
3. Work rumination	4.78	1.44	0.28***	0.34***	–							
4. OCB	3.59	0.59	0.43***	0.34***	0.28***	–						
5. Job crafting	3.52	0.57	0.20***	−0.06	0.00	0.11*	–					
6. Psychological capital	4.53	0.77	0.15**	−0.10*	0.09*	0.13**	0.16***	–				
7. Gender	–	0.50	0.08	0.01	−0.10*	0.06	−0.01	0.08	–			
8. Age	37.08	8.08	0.12**	0.04	−0.03	0.16***	0.03	0.05	0.00	–		
9. Marital status	–	0.47	0.08	0.04	−0.02	0.10*	0.02	−0.06	0.13**	0.44***	–	
10. Number of children	2.10	2.16	0.07	0.03	0.00	0.14**	0.02	−0.04	0.15***	0.60***	0.57***	–
11. Job tenure	7.86	6.40	0.02	−0.03	−0.05	0.07	0.05	0.05	0.08	0.63***	0.36***	0.47***

*N* = 523; WFC, work-to-family conflict; OCB, organizational citizenship behavior. Gender (1 = male, 2 = female) and marital status (1 = single, 2 = married) were categorically measured. Age and job tenure were measured in years. *p < 0.05, **p < 0.01, ***p < 0.001.

### Hypotheses testing

**Figure 2:**
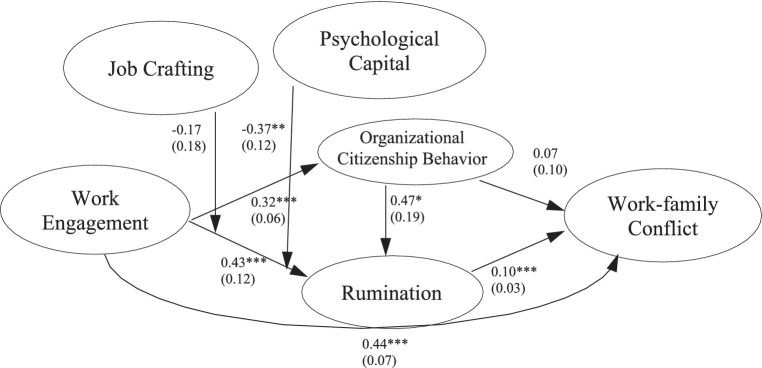
Results of structural equation modeling with latent variables. We report unstandardized coefficients and their standard errors in brackets. We depict only latent variables for reasons of clarity. *N* = 523. *p < 0.05, **p < 0.01, ***p < 0.001.

Taken together, Hypotheses 1 to 6 specify a model in which OCB and work rumination mediate the relationship between work engagement and work–family conflict, and in which psychological capital and job crafting buffer the relationship of work engagement with work rumination. We tested all six hypotheses using a single-structure equation model with latent variables (see Figure 2). The model fitted the data acceptably [*χ^2^* (482) = 956.28, CFI = 0.93, TLI = 0.92, RMSEA = 0.04; we obtained these fit values from a model without interactions, because models with interactions do not provide fit indices]. Results supported Hypothesis 1, as indicated by a positive relationship between work engagement and work–family conflict (*b* = 0.44, s.e. = 0.07, *p* < 0.001, 95% CI = 0.30, 0.58). In contrast to Hypothesis 2, OCB did not mediate the relationship between work engagement and work–family conflict (unstandardized estimate of the product-of-coefficients = 0.02, s.e. = 0.03, *p* = 0.47, 95% CI = −0.04, 0.09). However, in line with Hypothesis 3, results revealed that work rumination partially mediated the relationship between work engagement and work–family conflict (unstandardized estimate of the product-of-coefficients = 0.04, s.e. = 0.02, *p* = 0.01, 95% CI = 0.01, 0.07). Thus, data supported Hypothesis 3. Similarly, our results showed that OCB and work rumination sequentially (and partially) mediated the relationship between work engagement and work–family conflict (unstandardized estimate of the product-of-coefficients = 0.02, s.e. = 0.01, *p* = 0.03, one-tailed, 90% CI = 0.002, 0.03). Thus, results supported Hypothesis 4.

**Figure 3:**
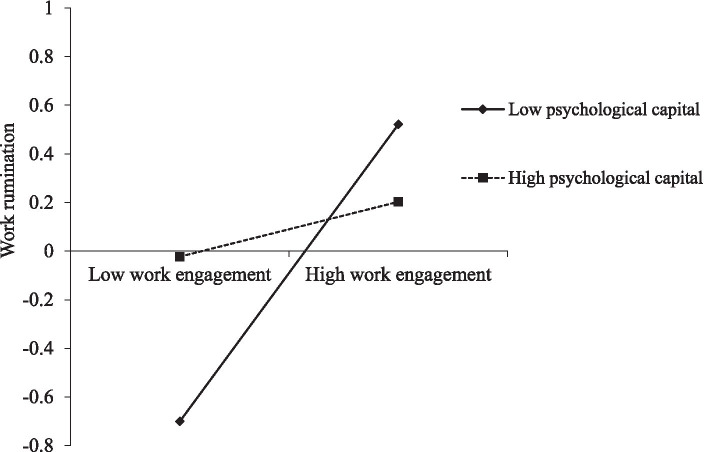
Psychological capital moderates the relationship between work engagement and work rumination.

Additionally, results did not support the buffering role of job crafting in the relationship of work engagement with work rumination (*b* = −0.17, s.e. = 0.18, *p* = 0.34, 95% CI = −0.52, 0.18), and thus, Hypothesis 5 was not supported. In contrast, results revealed a significant interaction of work engagement with psychological capital in predicting work rumination (*b* = −0.37, s.e. = 0.12, *p* < 0.01, 95% CI = −0.61, −0.13). Figure 3 depicts the simple slopes for 1 *SD* above and 1 *SD* below the mean of psychological capital. Work engagement was only significantly related to work rumination at low levels of psychological capital (*b* = 0.72, s.e. = 0.15, *p* < 0.001, 95% CI = 0.43, 1.00), but not at high levels (*b* = 0.13, s.e. = 0.16, *p* = 0.40, 95% CI = −0.18, 0.44). Thus, results supported Hypothesis 6.

## Discussion

Although high work engagement is typically perceived as highly desirable from the perspective of both employers and workers (e.g., Halbesleben et al., 2009; van Zyl et al., 2021), there is rising concern that work engagement is associated with certain negative consequences (Demerouti et al., 2015). We already know from existing literature that individuals who experience work engagement tend to invest personal resources in performing their jobs and engage in extra-role behavior (Halbesleben et al., 2009; Bolino et al., 2015). Our findings that work engagement is positively related to OCB support the resource investment principles of the COR model (Hobfoll et al., 2018) as individuals who have high work engagement are enthusiastic about their work and perform their work with vigor are likely to invest cognitive, emotional, and physical resources in performing extra role behaviors at work. However, when engaged employees exhibit OCBs, they may experience further resource loss beyond what they would have ordinarily lost performing job duties.

Our findings demonstrate that work engagement leads to high investment in one’s work that threatens investments in family roles. Our findings can be understood from the negative side of workaholism. Although workaholism which is viewed as pathological aspect of heavy work investment is distinct from work engagement which is seen as healthy work investment. However, unbalanced work engagement and workaholism are characterized by high intensity and working excessively due to the tendencies of both to exceed the usual working day limits (Di Stefano and Gaudiino, 2019). Workaholics and work engaged employees also are known to be highly absorbed in their work (Di Stefano and Gaudiino, 2019). This overlap in the characteristics of workaholism and work engagement (e.g., Gorgievski et al., 2010) suggests that they could separately lead to positive job outcomes such as work performance and OCB (Halbesleben et al., 2009; Gorgievski et al., 2010) as well to negative outcomes such as work–family conflict. This finding appears to support emerging evidence that positive constructs such as organizational identification noted to be beneficial to the organization may also have dark sides on the experience of satisfaction and work–family conflict (Irshad and Bashir, 2020). In line with the tenets of COR, it is possible that work engagement could be desirable at a certain level but detrimental at very high levels (Shimazu et al., 2018), especially when there is no deliberate effort to conserve resources or improve recovery experience. Future studies on the test of the resource model should include curvilinear relationships between work engagement and positive and negative individual outcomes, such as well-being and performance both at work and home.

Moreover, work engagement was positively related to high levels of work-related rumination and work–family conflict. In particular, our findings reveal that the positive relationship between OCB and work–family conflict is mediated by work-related rumination. Engagement in family activities after work requires investment of cognitive, energy, and time resources and this happens when the individual has adequate resources to invest in such activities. Rumination about work hampers effective handling of family responsibilities as work-related thoughts continue to interfere with thoughts about family. This finding extends prior studies assuming a direct relationship between OCB and work–family conflict (e.g., Bolino and Turnley, 2005) and other studies on the mediation effects of OCB on the engagement-work–family conflict relations (Halbesleben et al., 2009). This is in line with the principles of COR theory and the W-HR explanations of how investment at work may negatively affect family roles. As we observed in our study, work engagement enables an individual to go the extra mile of exhibiting OCB. Engagement in OCB involves investment of resources at work which makes employees vulnerable to rumination about work after work periods. Rumination in turn further consumes available resources and because resources are finite, individuals would not have enough resources to fulfill family roles resulting in work–family conflict.

The findings that psychological capital moderates the relationship between work engagement and work rumination is in line with our reasoning that psychological capital serves as a resource reservoir which employees draw from when performing work roles. Psychological capital was also negatively related to work–family conflict. This is because psychological capital is not only related to satisfaction and wellbeing (Avey et al., 2011; Luthans et al., 2015), and fosters positive emotions (Rosales, 2016), it also assists employees to experience superior performance and as such limits the extent to which they regurgitate work-related issues. Individuals with a strong psychological capital are also less affected by the negative situations they experience in their working life (Konkel and Heffernan, 2022) and helps employees to thrive and flourish (Biswal et al., 2023) and as such may inhibit employees from the negative effects of rumination.

This finding is also consistent with the propositions of the resource theory which posits that individuals who have psychological capital are more likely to mobilize new resources and these resources are reinvested when performing work roles (Hobfoll, 2011) and may still have enough resources to cope with demands outside the workplace which may have implications for the experience of work–family conflict. Our results showed that people high in psychological capital tend to ruminate more about work. This finding is intriguing as it shows that individuals with high psychological capital who likely have reinvested much time and energy resources in performing work roles may still have enough energy to invest in other activities and domains after work. As shown in our study, psychological capital buffers the effect of work engagement on rumination as work engagement was only significantly related to work rumination at low levels of psychological capital but not at high levels. Although we did not consider whether the participants were particularly ruminating about negative or positive work events which may be necessary to understand rumination outcomes (Gruber et al., 2011; Yang et al., 2020), our results suggest that psychological capital is important during work and after work periods. Our findings lend credence to the COR (Hobfoll, 2011) and W-HR (ten Brummelhuis and Bakker, 2012) views that personal resources such as psychological capital are not only important in protecting resource loss due to resource investment in one domain but will enable the individual to have enough resources to perform in other domains. Interestingly, our findings, especially the link between work engagement and work–family conflict are mostly consistent with the results of previous studies conducted in more developed countries of North America. Our finding that engagement is related to work interference with family suggest that work-family issues are also important in both developing countries, such as Nigeria, as it is in more developed countries. As family life is highly valued and sometimes above work life in Nigeria (Amazue and Onyishi, 2016; Ugwu et al., 2019), the negative impact of work engagement on family life may become more obvious in such an environment. A cross-cultural study involving samples from diverse cultures may help in clarifying our understanding of the contexts where the negative outcomes of work engagement is more likely to occur.

Our hypothesis on the moderating effects of job crafting was not confirmed. The result is inconsistent with earlier studies (e.g., Tims et al., 2013) which suggest that job crafting has both resource accumulation and conservation elements that may protect individuals from adverse resource loss during work. Lu et al. (2014) also found that work engagement is related to job crafting as engaged employees tend to craft their jobs in order to increase person-job fit. This means that job crafting may be important in responding to job conditions during work periods but may not necessarily lead to better adaptation after work as demonstrated in our study. In addition, some aspects of job crafting require investment of resources. For instance, the decreasing hindering job demands component of job crafting entails that the individual devout available personal and job resources to take actions that reduce negative job demands (Tims et al., 2012). In our study we treated job crafting as a composite, meaning that job crafting can help individuals to build resources and may also require investment of resources. The non-significant moderating effect of job crafting may be linked to the resource investment aspects of job crafting whereby employees invest resources crafting their job and may have limited resources to perform in other domains. The possible negative impact of job crafting including its interference with home activities has been reported in previous studies (Akkermans and Tims, 2017; Zito et al., 2019).

Finally, the non-moderating effects of job crafting may also be related to the work environment where the participants were drawn from. It has been demonstrated that employees engage in job crafting when there is an opportunity to do so (e.g., van Wingerden and Niks, 2017). Although we did not compare our sample with other samples from Western countries, opportunities for job crafting in Nigeria may differ from other countries. Onyishi and Ogbodo (2012) noted that employees in Nigeria appear not to take proactive measures to positively influence work outcomes indicating that there may be low motivation or limited opportunities for job crafting. Earlier studies (House et al., 2004) demonstrate that Nigeria differs from most Western cultures in terms of collectivism, power distance and performance orientation. In comparison with other countries in North America and Europe, Nigerians score higher in in-group collectivism and power distance and lower in performance orientation. In high power distance cultures such as Nigeria, employees perceive their bosses as superior and largely depend on them for decision-making at work, and are not likely to take initiatives to change their job situations and craft their jobs. In the same manner, individuals in low performance orientation and high collectivistic cultures are also not likely to bother about individual accountability in job performance and may engage in lower job crafting behaviors than employees in high performance orientation and low collectivistic cultures. Although job crafting might build resources, it also requires resources of employees particularly if it is not supported by the organization (leadership structure). To fully understand the role of job crafting in buffering the negative effects of work engagement in Nigeria, it may be important to understand job crafting opportunities in the country.

### Practical implications

This study highlights the concern that engaged workers could experience work–family conflict through engaging in OCB and/or work rumination. Managers of work organizations need to be aware of the detrimental effects of work engagement particularly among their highly motivated workers. As engagement in OCB is generally seen as positive organizational behavior because of its impact on performance it could be desirable to adopt strategies that may help employees continue to exhibit OCB but not experience its negative impact. After-work programs, such as leisure and relaxation, and other related programs targeted at helping employees switch off from work-related activities to other non-work activities could help employees ruminate less about work and experience less work–family conflict when they return home even when they may have been highly engaged and performed OCB during work periods.

In addition, our findings suggest the potential for managers to develop strategies for improving personal variables (psychological capital) that can buffer the effects of high work engagement on work rumination, which in the long run reduces the impact work engagement may exert on work–family conflict. Specifically, organizations may want to strengthen their employees’ psychological capital. There is evidence that organizations can successfully improve psychological capital through training and positive leadership behaviors (Luthans et al., 2006). This can be done through human resources management strategies such as psychological capital intervention (Luthans et al., 2006) which involves the development of the four aspects of psychological capital (hope, self-efficacy, optimism, and resilience) through a series of exercises and group discussions. Organizational leaders can also through their behavior create an environment that helps organizational members develop high psychological capital.

### Limitations and future research directions

This study has some limitations which provide fruitful avenues for future research directions. First, we collected all our data with self-report measures. This raises concerns regarding common methods bias (Podsakoff et al., 2012). However, we reduced such biases by distributing data collection across three measure times, where data for the independent variable, mediators, and outcome variable were collected at different points in time. In future research, however, the use of multiple sources of data and full longitudinal designs is desirable. Relatedly, we encourage future research to go beyond three waves of data and use an additional fourth measurement point in time to additionally separate the assessment of our two mediators work rumination and OCB. Thereby, future work would further alleviate concerns of common method bias and use a more rigorous study design to test our sequential mediation hypothesis.

Second, we only investigated the mediating role of work rumination and OCB on the relationship between engagement and work–family conflict and there could be other possible mechanisms that may account for this relationship. For instance, technology use after work could also mediate the relationship between work engagement and work–family conflict. Engaged employees may not only put extra effort into performing work roles during work, but they may also be more inclined to use technology at home to perform work-related activities. The use of technology at home has been found to lead to poor recovery from work as well as associated with work–family conflict (Park et al., 2011).

Third, we treated job crafting as composite. This might have undermined its moderating capabilities. It is reported that unique circumstances such as cultural differences and in-group collectivism can cause some dimensions of or different approaches to job crafting to change shape or form (Harju et al., 2021; Boehnlein and Baum, 2022) and may exert different effects. Based on this shortcoming, future research should examine the multidimensional nature of job crafting in the relationship between work engagement and work rumination.

Finally, we cannot rule out reciprocal relationships among our study variables, because we did not use a full longitudinal design where all variables of interest are assessed at each measurement point in time. For instance, high work engagement can lead to high work–family conflict due to resource loss as demonstrated in our study and high work–family conflict might as well reduce work engagement. We encourage future studies to address such reciprocal relationships.

## Conclusion

Work engagement is generally seen as positive organizational behavior because of its link with positive outcomes but there is emerging evidence to show that it could also have negative impacts, especially on after-work outcomes. Employees who experience high work engagement tend to devote a lot of time and energy at work performing job responsibilities which may make them have difficulties coping with family responsibilities after work periods. Performing family roles after work periods also requires investment of resources. The present study demonstrates that work engagement through OCB or work rumination can lead to work–family conflict. Rumination about work hampers effective handling of family responsibilities as work-related thoughts continue to interfere with thoughts about family leading to work–family conflict. As found in our study, the negative impact of work engagement on rumination could be reduced with interventions that increase employee psychological capital.

## Data availability statement

The raw data supporting the conclusions of this article will be made available by the authors, without undue reservation.

## Ethics statement

The studies involving humans were approved by Research Ethics Committee, Department of Psychology, University of Nigeria, Nsukka. The studies were conducted in accordance with the local legislation and institutional requirements. The participants provided their written informed consent to participate in this study.

## Author contributions

IO: Writing – review & editing, Writing – original draft, Visualization, Validation, Supervision, Software, Resources, Project administration, Methodology, Investigation, Formal analysis, Data curation, Conceptualization. CN: Writing – review & editing, Writing – original draft, Software, Methodology, Formal analysis, Data curation. FU: Writing – review & editing, Writing – original draft, Validation, Methodology, Investigation. LA: Writing – review & editing, Writing – original draft, Investigation. GH: Writing – review & editing, Writing – original draft, Supervision.
